# The Eco-Generativity Scale (EGS): A New Resource to Protect the Environment and Promote Health

**DOI:** 10.3390/ijerph20156474

**Published:** 2023-07-28

**Authors:** Annamaria Di Fabio, Andrea Svicher

**Affiliations:** 1 Department of Education, Languages, Intercultures, Literatures and Psychology (Psychology Section), University of Florence, via di San Salvi, 12, Complesso di San Salvi, Padiglione 26, 50135 Florence, Italy; 2 THE—Tuscany Health Ecosystem NextGeneration UE-NRRP, Department of Education, Languages, Intercultures, Literatures and Psychology (Psychology Section), University of Florence, via di San Salvi, 12, Complesso di San Salvi, Padiglione 26, 50135 Florence, Italy; andrea.svicher@unifi.it

**Keywords:** eco-generativity, eco-generativity scale, health, well-being, eco-anxiety, psychology of sustainability and sustainable development, sustainability science

## Abstract

(1) Background: Environmental issues are among society’s most pressing concerns as they can significantly impact the environment and human health. The Eco Generativity Scale (EGS), a 28-item four-factor scale has been introduced to promote a constructive outlook on the matter. It encompasses two types of generativity, namely ecological and social generativity, as well as environmental identity and agency/pathways. The aim of the current study was to examine the EGS’s psychometric properties among 375 Italian university students. (2) Methods: To evaluate the scale’s factor structure, both exploratory and confirmatory factor analyses were conducted. Internal consistency was evaluated via Cronbach’s alphas and McDonald’s omega. Concurrent validity was analyzed with the Positive and Negative Affect Scale (PANAS), Satisfaction with life Scale (SWLS), Meaningful Life Measure (MLM), and Flourishing Scale (FS). (3) Results: The exploratory factor analysis showed the best fit for a four-factor solution. Confirmatory factor analysis revealed that a four-factor higher-order model provided the best fit to the data with good internal consistency. Furthermore, each factor and the total score showed a good concurrent validity with the PANAS, SWLS, MLM, and FS. (4) Conclusions: The Eco-Generativity Scale (EGS) showed good psychometric properties for its use in research and intervention as a promising tool to measure eco-generativity.

## 1. Introduction

Nowadays, environmental challenges such as climate change, global warming, forest degradation, desertification, forest fires, lack of freshwater supplies, and decreasing biodiversity are among the most pressing issues for the twenty-first-century society and economy, as well as an important concern for human health [1,2]. In this scenario, the population’s health is being harmed in a variety of ways [3,4,5,6] with an increase in negative psychological states too [7]. As a result, the achievement of sustainability of life on Earth has emerged as one of the most compelling global scientific, political, and educational issues [8,9]. In the psychological literature, a fast-growing research perspective dealing with the study of “eco-anxiety”, as a psychological construct focused on the adverse effect of climate crisis, has been emerging [10]. According to Clayton, eco-anxiety is “a chronic fear of environmental doom” marked by concerns about climate activities and global warming crisis impacts [11]. Others psychological constructs associated with negative psychological states and environmental challenges are climate change worry [12], climate anxiety [13], environmental distress [14], ecological stress [15], and ecological grief [16], just to name a few.

More recently, Di Fabio and Svicher [17] have proposed a shift in the perspectives and approaches to contemporary research on the individual’s psychological experiences linked to environmental challenges. This perspective shift adheres to the principles of the psychology of sustainability and sustainable development [18,19,20,21], within the transdisciplinary Sustainability Science [22,23,24]; it emphasizes the meaning paradigm [25] and positive-oriented processes that renew resources rather than simply conform to principles aimed to cope with a declining supply of resources [26].

Therefore, the authors have introduced the new concept of eco-generativity as well as an instrument to measure it, namely the Eco-generativity Scale. Eco-generativity refers to the capacity of individuals to contribute to the preservation of the environment and promote sustainable practices for the benefit of future generations. It involves a sense of responsibility towards communities and the environment that ensures the continuity of life on Earth, passing on a healthy environment to future generations [17]. From this perspective, the construct of eco-generativity, on the one hand, follows the evolution of the broader construct of generativity [27] beyond the areas of research on personality [28], and on the other hand it expands the psychological processes enclosed in the concepts of generativity associated with the environment and the natural world [29,30,31].

In order to shed light on the evolution and enrichment of the construct of generativity in the scientific literature, the first aspect to address is Erikson’s contribution [27,32,33,34,35]. According to Erikson [27], generativity refers to a phase of personality development in which midlife adults, who have achieved a clear sense of self, can start to dedicate themselves to nurturing and guiding future generations. Furthermore, generativity deals with the capacity to provide a creation of the adult self, like a kid, or a piece of knowledge that is deliberately and unselfishly shared with others like a book, or an idea, created in order to leave something behind, encouraging generational continuity [27,35]. Subsequently, scholars have moved beyond the concept of a “generativity stage” and a phase that occurs within a specified period of the life span. Therefore, they have highlighted various aspects of generativity that can manifest in the individual’s personality from early to late adulthood [30,36,37,38].

McAdams et al. [36] define generativity as a two-step process of creating a product that extends one’s sense of self and relinquishes ownership of it to others. They proposed a multidimensional personality construct for generativity consisting of seven facets: cultural demand, inner desire, concern for future generations, belief in the goodness of humanity, generative commitment, generative action, and narration of generativity. These facets can appear in early, middle, or late adulthood and are arranged based on psychosocial demands and aligned with personal or cultural goals of providing for future generations [36]. In this framework, generativity can be conceived in different forms such as biological (e.g., nurturing children), parental (including providing for and disciplining one’s children), technical (involving transferring skills to those with less proficiency, often implemented by teachers), and cultural (wherein teachers transmit not only skills but also their meanings) [39]. Other scholars and researchers have further expanded the idea that generativity could overtake an age-ordered, phase-based construct, illustrating that generativity could involve those younger than midlife individuals [40,41]. Lawford et al. [40] observed that generativity is an aspect of moral concern in early adulthood. Komives et al. [41] found that generativity was the fifth of six stages in college students’ leadership identity development model (LID). Currently, in literature, there is a growing consensus that the construct of generativity could be observed across the whole adult lifespan [42,43]. Studies on generativity thus showed interindividual differences among different ages, from adolescence to older adulthood [44,45]. Moreover, in line with Frensch et al. [46] and McAdams and Logan [47], generativity could be observed in different environments, such as family life, working activities, volunteering, and activism. Therefore, the enrichment of the application of the generativity’s construct is also extended by researchers to the environmental challenges [29,30,31]. McAdams and de St. Aubin [30] identified ecological issues among the generative concerns as sources of motivation for stimulating environmental actions in favor of future generations; however, they did not further expand this approach. Schoklitsch and Baumann [31] furnished a first outlook on a new form of generativity extended to the environment (ecological generativity); nevertheless, they did not consider it as a construct but as the third factor of a broader measurement model for older adults, together with Kotre’s [39] four forms of generativity. Alisat and colleagues [29] investigated the links between generativity and individual response to environmental issues, observing that generativity was positively associated with environmental identity, environmental narratives, and strong feelings of connection with nature. Nevertheless, the authors did not expand their results into a new construct. Therefore, the lack of a clear conceptualization and the absence of a multi-dimensional operationalization of eco-generativity may have limited the research and understanding of the construct.

Starting from these bases, eco-generativity [17] was advanced as an integrated construct that builds on previous research. It includes four facets: two forms of generativity, ecological generativity and social generativity, as well as environmental identity and a facet concerning the perception of being able to attain goals by creating successful plans (Agency/Pathways). The ecological generativity facet has its foundation in the key concept of Schoklitsch and Baumann [31], enriching it with the most recent advancement in generativity research that suggests going beyond an age-ordered perspective. Thus, in this line, the generativity facet has been released from age’s restriction. It is an aspect of eco-generativity because it includes several concerns for the environment as motivational sources for leading people towards generative and pro-environmental actions [30]. The social generativity facet is grounded in the concept of social generativity [48,49,50] and it is an aspect of eco-generativity since it encloses responsibility and prosocial attitudes for future generations, considering the impact of individual behaviors on the community’s future. Both ecological generativity and social generativity facets include a future-time perspective of care for environment (ecological generativity) and people (social generativity) for future generations; this also includes engagement in activism to preserve the health of environment and communities [30,48,49,50]. The Environmental Identity factor draws upon the valuable concept of environmental identity [51] and deals with the feeling of being connected with aspects of the non-human natural world. Environmental identity is part of eco-generativity since generativity, environmental concerns, and concerns associated with the natural world were strongly associated and mutually influential [29,52,53]. The Agency/Pathways factor is composed of interrelated elements of a sense of successful determination in meeting goals in the past, present, and future (Agency) and a sense of being able to generate effective plans to meet goals (Pathways) on the basis of the construct of Hope [54]. They are part of ecological generativity according to McAdams and de St. Aubin [30] that illustrated positive motivational aspects of confidence and of success in the future as necessary elements to shift from generativity concerns to generative commitment and actions [30].

Eco-generativity differs from other concepts or measures of environmental attitudes or behaviors, such as environmental concern, pro-environmental behavior, environmental values, and environmental attitudes. Environmental concerns have been conceptualized in different ways. The first conceptualizations deal with responsibility and taking care of the environment, not to save the natural world itself but out of concerns about the environmental degradation of health and well-being [55,56]. Subsequent conceptualizations refer to concerns about the severity of environmental issues, knowledge of how human behavior affects them, and support for solutions [57,58]. Eco-generativity is distinct from environmental concerns since it encompasses protecting the environment and communities not for health and well-being concerns but for selflessly leaving a healthy world as a legacy and promoting life continuity. Moreover, eco-generativity, rather than focusing on the negative effect of human behavior on the environment and solutions for solving ecological issues, is focused on ecological and social generativity accompanied by the ability and awareness to achieve goals (Agency/Pathways) which are anchored to environmental identity. Pro-environmental behaviors refer to behaviors that consciously contribute to protecting the natural world and improving environmental sustainability [59,60,61]. Therefore, eco-generativity could be considered an antecedent of pro-environmental behaviors and could reinforce them. The same could be true for sustainable consumption, which brings together concepts related to environmentally friendly conduct in the context of acquiring services and products [62]. Eco-generativity could be considered as one of the antecedents of sustainable consumption and could strengthen them. For example, individuals high in eco-generativity could have a higher awareness of being engaged in pro-environmental behavior to leave a healthy environment to future generations, also perceiving themselves as able to achieve these goals in line with their environmental identity. Environmental values are conceived within Schwartz’s theory [63,64] and differentiated between biospheric-altruistic value (giving importance to other species, ecosystems, and the whole biosphere), humanistic-altruistic values (assigning weight to others until it encompasses all humanity), and self-interest values (considering solely the consequences for personal and close kin) [65]. Eco-generativity is different from environmental values since it is a personality-related construct composed of beliefs, emotions, and behaviors associated with both ecological and social generativity, pathways/agency, as well as environmental identity. Since eco-generativity encompasses ecological generativity and environmental identity, it could be positively related to biospheric-altruistic value, and since it includes social generativity, it could be positively related to the values of humanistic altruism. In a different way, eco-generativity could be negatively associated with self-interest values. According to Hawkcroft and Milfont [66] pro-environmental attitudes are described as a person’s propensity to show favor for the environment. Eco-generativity encapsulates ecological generativity and environmental identity. Ecological generativity is distinct from pro-environmental attitudes since it refers to exhibiting a favor toward the natural world for future generations, and environmental identity includes a propensity for the environment that originates from feeling part of the natural world. Therefore, eco-generativity and pro-environmental attitudes could be considered correlated. Eco-generativity is also different from environmental self-efficacy [67], which is a domain-specific form of self-efficacy [68] in pro-environmental behaviors (e.g., recycling or electricity saving). Eco-generativity differs from environmental self-efficacy because it includes the intertwined agency/pathways cognitive dimension, which encloses a goal-directed determination (agency) and planning ways to achieve objectives (pathways). It is a positive enduring disposition in terms of hope, consistent across situations and time, and it could be applied in different settings. This general positive disposition involving the determination to achieve goals is crucial for paying attention to future generations and to potentially acting for them and the environment [36]. Furthermore, self-efficacy and hope are structurally distinct in the literature [69]. Again, eco-generativity and environmental self-efficacy could be considered correlated.

Starting from these premises, the scale was developed also to facilitate new opportunities through eco-generativity, considering virtuous circles of mutual interaction between prosocial behaviors, pro-environmental behaviors, and sustainable developments to promote well-being and protect against adverse psychological states such as eco-anxiety [17]. Accordingly, the Eco-Generativity scale comprises 28 items and four factors rated on a 7-point Likert scale that reflects the above-mentioned four facets. The Ecological Generativity factor is rooted in the valuable concept of ecological generativity [31] which includes taking care of the energy used, respecting the environment, and living sustainably, including protecting animals.

The Social Generativity factor is rooted in the concept of social generativity [48,49,50] and encompasses taking care of future generations and recognizing the influence of one’s activities on the future of the community. Environmental Identity factor is rooted in the construct of environmental identity [51] that pertains to the recognition of oneself as a part of the natural world, the allocation of efforts and resources towards preserving the environment, the adoption of sustainable habits, and the sense of calmness and connectedness experienced in natural settings. Lastly, the Agency/Pathways factor is composed of interrelated elements of a sense of successful determination in meeting goals (Agency) and a sense of being able to generate successful plans to meet goals (Pathways) on the basis of the construct of Hope [54]. It emphasizes cognitive appraisals of goal-related capabilities including goal attainment, a focus on success rather than failure, a sense of challenge, and a relatively positive emotional state during goal-related activities [17].

The Eco-Generativity Scale showed preliminary findings for a four-factor solution [17]. However, the previous study on its psychometric properties did not investigate the Eco-Generativity Scale via confirmatory factor analysis leaving the question of the latent structure of the construct open. The aim of the present study is to examine the psychometric properties of the Eco-Generativity Scale via both exploratory and confirmatory factor analyses in university students. Internal consistency (i.e., reliability) and concurrent validity were also examined.

## 2. Materials and Methods

### 2.1. Participants and Procedure

The current study was conducted on 375 university students from Central Italy (Females = 62.7%; males = 37.3%; mean age = 20.58 years; SD = 3.08). They were recruited on a voluntary basis and provided informed consent according to privacy Italian laws (DL-196/2003; EU 2016/679). The administration order of applied questionnaires was balanced to contain the presentation order effects.

### 2.2. Measures

The Eco-Generativity Scale (EGS) [17] is a 28-item scale with answers rated on a seven-point Likert scale (ranging from 1 = strongly disagree to 7 = strongly agree). The EGS consists of four factors: Ecological Generativity, Social Generativity, Environmental Identity, and Agency/Pathways factor. The reliability of the scale was reported in the results section of the current study.

The Positive and Negative Affect Schedule (PANAS) [70]—Italian version [71] is a self-report scale composed of 20 adjectives evaluated by participants on a five-point Likert scale (ranging from “Very slightly or not at all” to “Extremely”). Ten adjectives compose the Positive Affect scale (PA) (enthusiasm, interest, determination), while the other ten are included in the Negative Affect scale (NA) (nervousness, fear, anguish). Cronbach’s alpha for PA was 0.83, whereas, for NA, it was 0.88.

The Satisfaction with Life Scale (SWLS) [72]—Italian version [73]—is a unidimensional five-item self-report scale that measures cognitive processes related to the overall subjective perception of well-being, focusing on the individual’s autonomous judgment capacity [72,73]. Respondents answered items on a seven-point Likert scale (ranging from “Strongly agree” to “Strongly disagree”). Cronbach’s alpha was 0.86.

The Meaningful Life Measure (MLM) [74]—Italian version [75] is a 23-item self-report questionnaire that measures five dimensions and a total score of life meaning on a 7-point Likert scale (ranging from “Strongly disagree” to “Strongly agree”). The five dimensions are Accomplished life (feeling personal goals are achieved), Principled life (having a personal philosophy or framework for understanding life), Purposeful life (having specific aims and ambitions), and Valued life (framing a sense of the intrinsic value of life). The total score of the scale was used in the current investigation, showing excellent Cronbach’s alpha (α = 0.91).

The Flourishing Scale (FS) [76]—Italian version [77]—is an 8-item self-report scale that measures sociopsychological prosperity related to perceived success in relevant areas of the individual’s life, such as self-esteem, relationships, and optimism [76,77]. Respondents indicate their degree of agreement on a 7-point Likert scale (ranging from “Completely disagree” to “Strongly agree”). Cronbach’s alpha was 0.89.

### 2.3. Statistical Analysis

Exploratory (EFA) and confirmatory factor analyses (CFA) were conducted by applying weighted least squares means and variance adjusted (WLSMV) in the lavaan 0.6-15 R package. For the EFA, the number of factors to retain was defined according to Kaiser’s eigenvalue criterion (eigenvalues > 1). All the solutions comprised from one and the number obtained via the Kaiser’s eigenvalue criterion were conducted with WLSMV estimation and Varimax rotation and were compared via the comparative fit index (CFI) and root mean square error of approximation (RMSEA). Item loading > 0.40 was considered good [78]. CFA was conducted to test three models: unidimensional (all items load on a single Eco-generativity factor), correlational (four correlated factors, namely Ecological Generativity, Social Generativity, Environmental Identity, Agency/Pathways), and higher order (the four factors are regressed onto a second Eco-generativity higher-order factor). Models were analyzed considering the comparative fit index (CFI), the Tucker–Lewis fit index (TLI) and root mean square error of approximation (RMSEA). CFI and TLI values > 0.97 were considered good, whereas values between 0.95 and 0.97 were considered acceptable. RMSEA values were considered good (≤0.05), adequate (between 0.05 and 0.08), mediocre (from 0.08 to 0.10), and unacceptable (>0.10) [79]. The reliability of the Eco-Generativity Scale was evaluated by means of Cronbach’s alphas (α) and McDonald’s omega (ω) using the Psych 2.3.3 R package. Values of α and ω > 0.70 were judged as adequate. Pearson’s correlation coefficients were used to calculate concurrent validity among the Eco-Generativity scale and Positive and Negative Affect Schedule, Satisfaction with Life Scale, Meaningful Life Measure, and The Flourishing Scale. The Psych 2.3.3 R package was used. All the analyses were implemented by means of R studio 2022.12.0 for Macintosh, Posit Software, Boston, MA, USA.

## 3. Results

The exploratory factor analysis (EFA) with WLSMV and Varimax rotation yielded four eigenvalues greater than 1. The first eigenvalue was 5.110, explaining 19% of the variance; the second was 3.014, explaining 11% of the variance; the third was 2.953, explaining 11% of the variance; and the fourth was 1.935, explaining 7% of the variance. As a result, we performed and compared 4-factor, 3-factor, 2-factor, and 1-factor solutions and found that the 4-factor solution had the best fit to the data (Table 1).

Therefore, we proceeded to examine the loadings of the 4-factor solution. The EFA results for the 4-factor solution conducted with WLSMV and Varimax rotation are presented in Table 2. All the ecological generativity items are loaded on the first factor, and all the social generativity items are loaded on the second factor. Similarly, all the environmental identity items are loaded on the third factor, and all the Agency/Pathways items are loaded on the fourth factor. Furthermore, all the items had good factor loadings higher than 0.40 on their respective factors.

**Figure 1:**
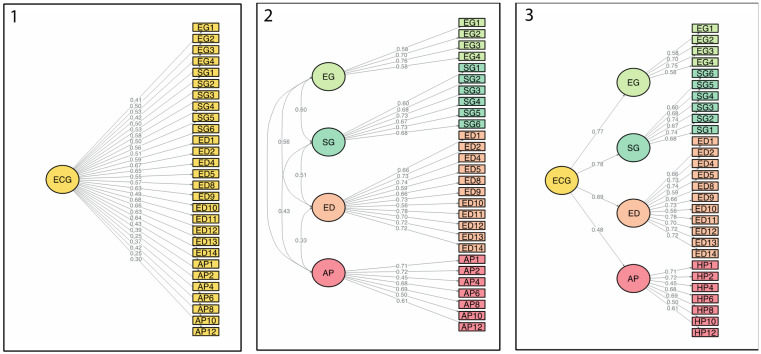
The Eco-generativity Scale: Path diagram of the tested models (Confirmatory Factor Analysis with Weighted Least Square Mean and Variance Adjusted [WLSMV]) (*n* = 375). 1 = 1-factor model; 2 = correlated four-factor model: 3 = four-factor higher order model. ECG = Eco-generativity; EG = Ecological generativity; SG = Social generativity factor; ED = Environmental Identity factor; AP = Agency/Pathways.

We then performed confirmatory factor analysis (CFA) comparing unidimensional, correlational, and higher-order models. The higher-order solution provided the best fit to the data, reflecting four factors (Ecological Generativity, Social Generativity, Environmental Identity, and Agency/Pathways) and an Ecological-generativity higher-order factor (Table 3). Path diagrams of the three tested models via CFA are shown in Figure 1.

We also calculated the reliability coefficient for both factors and the Ecological-generativity higher-order factor, reporting good values of Cronbach’s alphas and McDonald’s Omega (Table 4).

Finally, we analyzed the correlations between the Eco-Generativity Scale and the Positive and Negative affect scale (PANAS), the Satisfaction with Life Scale (SWLS), the Meaningful Life Measure (MLM), and the Flourishing Scale (FS). The four factors and the higher-order factor of the Eco-Generativity Scale showed positive and statistically significant correlations with PANAS PA, SWLS, MLM, and FS. Moreover, they showed negative and statistically significant correlations with PANAS NA (Table 5).

## 4. Discussion

The aim of the current study was to explore the psychometric properties of the recently advanced Eco-Generativity Scale using both explorative and confirmatory factor analysis. The factor structure of the scale was thus evaluated using both explorative factor analysis (EFA) and confirmatory factor analysis (CFA). Compared to the previous study on Eco-Generativity Scale [17], the current study offers the first results obtained via the CFA and correlations with two additional measures of well-being (PANAS and MLM). In scale development, the benefit of CFA over EFA is that CFA offers empirical indices of model fit (so that models can be compared) and the possibility to verify the predicted relationships between variables (e.g., relationships between an item and the latent factor with one subscale but not another) [80]. Expanding the knowledge on the relationship between eco-generativity and well-being is crucial for the psychology of sustainability and sustainable development since it is focused on the well-being of individuals and of the environment/s [20]. The explorative factor analysis (EFA) results indicated that four factors best fit the data, consistent with previous research conducted on Italian university students. Furthermore, confirmatory factor analysis (CFA) found that the higher-order model had the best fit for the data, indicating the presence of a higher-order Eco-generativity factor and four specific factors: Ecological Generativity, Social Generativity, Environmental Identity, and Agency/Pathways. This is consistent with the theoretical framework proposed by Di Fabio and Svicher [17], which posits a construct of eco-generativity encompassing these four concepts. Furthermore, the reliability of the three dimensions and the total score were found to be adequate, and the scale showed concurrent validity with measures of hedonic well-being (measured by the Positive and Negative Affect Schedule and the Satisfaction with Life Scale) and eudaimonic well-being (measured by the Meaningful Life Measure and the Flourishing Scale). These findings suggest that eco-generativity could be a promising variable for studying adaptive psychological responses to environmental challenges [17] and indicated that the Eco-generativity scale is a reliable instrument for assessing the construct. In this line, the application of the construct of eco-generativity could be promising for strength-based preventive perspective actions [81] aimed at fostering trainable psychological resources in individuals [82] to cope with the challenges of the XXI century, that also include environmental challenges. Concerning the organizational levels, eco-generativity could be a new psychological resource in the healthy organizations approach [83,84,85] which implements strength-based preventive perspective actions in organizations to promote healthy work environments, healthy business, and sustainable development [18,26]. Through this approach, eco-generativity could represent a construct for enriching the study and the application of the virtuous circle between positive psychological resources and psychological coordinates of sustainable development to promote the health and wellbeing of individuals and their environments, fostering positive connections between people and the natural world to assist sustainability efforts and wellbeing itself [18,20,21,26]. To strengthen this perspective, future cross-cultural validations of the scale are recommended to adapt the Eco-generativity Scale to a broader variety of countries, including Eastern and developing countries.

Although the results of the present study test the psychometric properties of this scale, some limitations need to be considered. The participants were university students from central Italy. Future studies could consider university students from different geographical areas in Italy, and from other international contexts. In addition, future research could be addressed to different participants, such as adult workers [86], older individuals [87] and vulnerable workers [88]. Moreover, additional studies could also refine the psychometric properties of the scale using Item Response Theory Models, which could help reduce the number of items [89,90] for a shorter version of the scale. It is in accordance with the accountability perspective, which encourages researchers to use evidence-based methodologies to ensure a balance in terms of cost-effectiveness [91,92]. Future studies could also investigate the study of the association between the eco-generativity and positive psychological constructs, exploring, for example, relationships with promising variables such as humor [93], courage [94], resilience [95], emotional intelligence [96], as well as critical individual differences such as perfectionism [97]. Finally, future studies on Eco-Generativity Scale could expand the knowledge on the nature of the construct by implementing qualitative analyses [98], for example, by administering open-ended questions together with the self-report scale. It could enrich the objective results obtained via a validated and trustworthy instrument with the subjective point of view of participants [99].

## 5. Conclusions

Even with the above-mentioned limitations, the Eco-Generativity Scale emerged as a reliable tool for accurately detecting eco-generativity. The development of this instrument could facilitate new research and intervention perspectives focusing on Eco-Generativity in strength-based prevention approaches [81]. Helping individuals and workers to cope with environmental concerns and sustain positive psychological processes related to the psychology of sustainability and sustainable development [20,21,26] could mean making a contribution to the health of the environment/s, individual/s, worker/s, as well as improving healthy organizations [17,83] and taking care of the planet and the new and future generations.

## Figures and Tables

**Table 1:** The Eco-generativity Scale: Fit indexes of Explorative Factor Analysis with Weighted Least Square Mean and Variance Adjusted (WLSMV) estimator (*n* = 375).

# of EFA Factors	Chi-Square (df)	*p*	CFI	RMSEA
4-factor	510.647 (272)	<0.001	0.998	0.027
3-factor	3736.471 (297)	<0.001	0.996	0.038
2-factor	1531.237 (323)	<0.001	0.946	0.075
1-factor	2290.753 (350)	<0.001	0.863	0.107

EFA = Explorative Factor Analysis; CFI = Comparative Fit Index; RMSEA = Root Mean Square Error of Approximation.

**Table 2:** Eco-generativity Scale: Factor loadings of Exploratory Factor Analysis with Weighted Least Square Mean and Variance Adjusted (WLSMV) (*n* = 375).

			1	2	3	4
# of Items	Item	Item Content	λ	λ	λ	λ
1	EG1	Being parsimonious with energy	0.561 *			
2	EG2	Preserving the environment	0.633 *			
3	EG3	Having an ecological way of life	0.681 *			
4	EG4	Minimizing rubbish	0.440 *			
5	SG1	Acting for a better world for younger people		0.683 *		
6	SG2	Being active in improving one’s own neighborhood.		0.656 *		
7	SG3	Relinquishing personal comfort to promote future generations’ development		0.724 *		
8	SG4	Guaranteeing the well-being of future generations.		0.582 *		
9	SG5	Being committed to leaving products of self that live longer than me		0.624 *		
10	SG6	Helping in individuals’ flourishing		0.457 *		
11	ED1	Experiencing time in natural surroundings			0.568 *	
12	ED2	Recognizing myself as part of the natural world			0.607 *	
13	ED4	Staying in contact with nature to cope with distress			0.726 *	
14	ED5	Feeling similarities with nature’s creatures			0.498 *	
15	ED8	Living in a house in natural surroundings when possible			0.716 *	
16	ED9	Living a worthy existence in touch with nature			0.777 *	
17	ED10	Enjoying the beauty of the natural world as artworks			0.563 *	
18	ED11	Feeling restored by staying in nature			0.792 *	
19	ED12	Being a keeper of the environment			0.532 *	
20	ED13	Feeling at ease in the natural environment			0.735 *	
21	ED14	Enjoying pleasure in finding natural settings in urban environments			0.654 *	
22	AP1	Finding different ways to go beyond a stagnant situation				0.596 *
23	AP2	Achieving objectives with energy				0.715 *
24	AP4	Solving problems in a variety of ways				0.456 *
25	AP6	Recognizing plenty of ways to fulfill my life’s goals				0.690 *
26	AP8	Effectively solving problems also when others give up				0.576 *
27	AP10	Living rather successfully				0.540 *
28	AP12	Achieving my objectives				0.694 *

λ: Standardized loadings; * = significant at 1% level.

**Table 3:** The Eco-generativity Scale: Fit indexes of Confirmatory Factor Analysis with Weighted Least Square Mean and Variance Adjusted (WLSMV) estimator (*n* = 375).

Models	Chi-Square (df)	*p*	CFI	TLI	RMSEA [95%CI]
1-factor	2372.181 (350)	<0.001	0.863	0.853	0.103 [0.099–0.097]
4-factor correlated	545.916 (344)	<0.001	0.984	0.982	0.036 [0.031–0.041]
4-factor higher order	588.759 (348)	<0.001	0.987	0.986	0.032 [0.027–0.037]

1 = 1-factor model; 2 = correlated four-factor model: 3 = four-factor higher order model.

**Table 4:** Cronbach’s alphas and Mc Donald’s Omega for the four-factor higher order model (*n* = 375).

Factors	Cronbach’s Alpha	Mc Donald’s Omega
EG	0.739	0.749
SG	0.839	0.842
ED	0.909	0.912
AP	0.816	0.820
ECG	0.908	0.911

EG = Ecological Generativity factor; SG = Social Generativity factor; ED = Environmental Identity factor; AP = Agency/Pathways factor; ECG = Eco-Generativity factor.

**Table 5:** Pearson’s correlations between the Eco-Generativity Scale, The Positive and Negative affect scale, the Satisfaction with Life Scale, the Meaningful Life Measure, the Flourishing Scale (*n* = 375).

	PANAS PA	PANAS NA	SWLS	MLM	FS
EG	0.113 **	−0.087 *	0.267 **	0.160 **	0.271 *
SG	0.199 **	−0.232 *	0.296 **	0.264 **	0.272 **
ED	0.191 **	−0.262 *	0.237 **	0.274 **	0.268 **
AP	0.580 **	−0.246 **	0.491 **	0.573 **	0.458 **
ECG	0.353 **	−0.187 *	0.354 **	0.454 **	0.320 *

EG = Ecological Generativity factor; SG = Social Generativity factor; ED = Environmental Identity factor; AP = Agency/Pathways factor; ECG = Eco-Generativity factor. PANAS PA = Positive Affect Negative Affect Schedule—Positive Affect; PANAS NA = Positive Affect Negative Affect Schedule—Positive Affect; SWLS = Satisfaction with Life Scale; MLM = Meaningful life Measure; FS = Flourishing Scale. ** *p* ≤ 0.01 * *p* ≤ 0.05.

## Data Availability

The data presented in this study are available from the corresponding author on reasonable request. The data are not publicly available due to privacy reasons.
